# Legal Status of Veterinary Colleges in New York City

**Published:** 1883-01

**Authors:** 


THE LEGAL STATUS OF VETERINARY COLLEGES IN NEW YORK CITY.—During the last session of the Legislature a bill passed both Senate and Assembly and received the signature of the Governor, which forever settles this question of the legal status of colleges chartered or incorporated under the General Laws of 1848.

We print the bill entire so that all may comprehend that *additional validity* is given (if any was needed, which we deny) to the charters of the institutions which have done so much and are still doing such excellent work in the advancement of veterinary science.

AN ACT to restrict the formation of corporations under an act entitled "An act to provide for the incorporation of benevolent, charitable, scientific and missionary societies," being chapter three hundred and nineteen of the laws of eighteen hundred and forty-eight, and the acts amendatory thereof, and to legalize the incorporation of certain societies organized there under, and to regulate the same.

*The People of the State of New York, represented in Senate and Assembly, do enact as follows:*

SECTION 1. Hereafter no literary or scientific college or university shall be incorporated under the provision of an act entitled "An act to provide for the incorporation of benevolent, charitable, scientific and missionary societies," being chapter three hundred and nineteen of the laws of eighteen hundred and forty eight and the acts amendatory thereof, without the approval of the regents of the university of the State of New York to be endorsed upon and filed with the certificates of incorporation, and the said regents, as a condition of such approval, may impose such conditions as in their judgment they shall deem advisable, which shall not conflict with said acts.

SEC. 2. All scientific and all literary colleges and universities, organized under said act, which shall have reported to the said regents within the two years last past are hereby declared legally incorporated, and all degrees heretofore and hereafter conferred by them are declared valid, and all such colleges and universities shall be subject to the same duties, obligations and liabilities, and to the same control and visitation of said regents, as colleges and universities chartered by said regents.

SEC. 3. All acts and parts of acts inconsistent herewith are hereby repealed.

SEC. 4. This act shall take effect immediately.

